# A Communication

**Published:** 1857-10

**Authors:** S. Ramsey

**Affiliations:** Reedsburg, Wis.


REEDSBURG, WIS., *Sept.* 17, 1857.

DR. N. S. DAVIS,—A case has occurred in my practice of so singular a nature, that I thought it might interest the readers of your Journal.

I was called the 13th of the present month to see a child that had just been born, which presented so singular an appearance that the midwife in attendance desired that a medical man should see it.

When I arrived at the house, I found the child had been born about half an hour previous, and upon examination, found that the child, a boy, was perfect every way excepting an entire want of development of the parieties of the abdomen. The chest was well developed, and the little fellow was crying lustily.

When born, there was nothing but the peritoneum to keep the viscera, the stomach, the liver, spleen and the intestines in their position; but this was soon afterwards ruptured by the child drawing up its knees, when all the viscera mentioned were exposed to sight. I turned the folds of the peritoneum aside, and found everything fully developed, save an entire want of muscular tissue from the inferior part of the chest to the pubes, and from spine to spine of the ilium. There was no umbilical cord, but the umbilical artery and vein were inverted in a thickened portion of the peritoneum.

The child had a passage of meconium while there, took food, and was apparently without pain, but died the next day, living about thirty hours from the time I saw it.

As to the cause of this strange phenomenon it would be folly to speculate. It might, however, be proper to state, that the mother is a healthy woman, with a family of eight children. Her husband died about four months ago, or about the fifth month of pregnancy, and during his sickness (measles) the entire family save herself were helpless with the same disease.

This condition of her family imposed a tax for two or three weeks more than her physical powers could bear, and added to the death of her husband, also being among entire strangers (they having just emigrated from Maine), was a source of mental anxiety which may possibly have had some connection with the immature condition of the child. But, as I before remarked, it is folly to speculate, and I merely give the facts for what they are worth. Respectfully,

S. RAMSEY.

